# Migration behavior and performance of the great spotted cuckoo (*Clamator glandarius*)

**DOI:** 10.1371/journal.pone.0208436

**Published:** 2019-01-04

**Authors:** Juan Diego Ibáñez-Álamo, Josse Rühmann, Tomás Pérez-Contreras, Manuel Soler

**Affiliations:** 1 Groningen Institute for Evolutionary Life Sciences, University of Groningen, Groningen, The Netherlands; 2 Departamento de Zoología, Facultad de Ciencias, Universidad de Granada, Granada, Spain; Charles University, CZECH REPUBLIC

## Abstract

The study of brood parasitism has traditionally been focused on the breeding period, but recent evidence suggests that it urgently needs a new spatio-temporal perspective to explore novel avenues on brood parasite-host co-evolutionary interactions. Many brood parasites are migrants, but their ecology outside their short breeding season is poorly known. The great spotted cuckoo (*Clamator glandarius*) is one of the classical models in the study of brood parasitism, however, there is very little information on its migratory strategy, route and wintering grounds. Furthermore, there is no previous information on the geographical distribution of mortality and its causes in this species; information that is critical to understand the fluctuations in cuckoo populations and detect potential conservation risks. Using satellite tracking technology, we provide novel insight into the migratory behavior and performance of the great spotted cuckoo. We found individuals from southern Spain to be long-distance nocturnal migrants that use the East Atlantic Flyway for both post and pre-breeding migration, and that winter in the western Sahel. We found evidence of individual variation in their migration route, particularly regarding their post-breeding behavior in Spain. Our study also suggests that the south of Morocco is the most dangerous area due to a large number of deaths during the post-breeding migratory period. Furthermore, we found that natural predation seems to be the main cause of death, probably due to raptors, although human activities (i.e. hunting) could also played a role in the southern Mediterranean shore. Our study offers novel findings and challenges traditional ideas on the ecology of this species providing a good example of how the new spatio-temporal perspective can expand our knowledge on brood parasites.

## Introduction

Brood parasitism is a strategy by which one species (the parasite) lays its eggs in the nest of another species (the host) whose adults provide parental care for the parasitic young [1]. Since the costs for the hosts are usually high, brood parasitism has led to an evolutionary arms-race between parasites and their hosts [2,3]. These brood parasite-host systems have significantly advanced our understanding of coevolution [1–3], but it has been recently suggested that we urgently need to incorporate a new spatio-temporal dimension to better understands these evolutionary interactions [4].

The study of brood parasites’ movements (i.e. migration) is one of the key features in this new spatio-temporal dimension [4]. Movement ecology is a fundamental discipline within spatial ecology [5] that has received increasing attention, particularly regarding birds, mainly due to recent advances in tracking technology [6,7]. Different aspects of movement ecology of brood parasites, like home ranges or territoriality, have attracted attention from researchers since the 1980s (e.g. [8–10]). However, these studies were still mainly restricted to breeding areas and short time (breeding) periods, and in many cases without marked individuals (e.g. [8,11–13]). Only recently some studies using new satellite tracking technology have started to follow individual brood parasites year-round, providing novel information on different aspects of their movement ecology such as their migration strategy [14,15] or home ranges [16].

Migratory behavior is generally important to understand the evolution of range limits, life-history trade-offs and population dynamics [17,18]. In addition, for brood parasites, it is also key to understand the spatial and temporal allopatry with their hosts. For instance, some studies have proposed that some brood parasites might be breeding both in their ‘typical’ breeding grounds and in their wintering areas (e.g. [12]). This hypothesis would suggest that host’s nest availability would be an important resource driving the migration of brood parasites, which could have important consequences for the coevolutionary relationship with their hosts, especially if they are parasitizing different species in each area [19]. Additionally, spatio-temporal patterns of mortality, which can be tightly related to migration behavior [20–22], are very important to understand population dynamics in the breeding areas [15]. Ultimately, they can further our knowledge on brood parasite-host relationships [4], as they will offer insights into potential selective pressures (i.e. predation or food availability) acting on brood parasites. Many brood parasites are migrants [23], but detailed information on their migratory behavior (i.e. their migratory routes and wintering grounds) or performance (i.e. mortality) is still scarce and mainly restricted to the common cuckoo (*Cuculus canorus*) [14,15,24–26].

The great spotted cuckoo (*Clamator glandarius*) is one of the three brood parasitic species, in addition to the common cuckoo and the Brown-headed cowbird (*Molothrus ater*), that has attracted the majority of previous spatio-temporal studies [4]. The great spotted cuckoo’s range spans southern Europe and sub-Saharan Africa [27] with Spain holding the largest proportion of breeding individuals (>80%) in Europe [28]. While the populations in sub-Saharan Africa are thought to be sedentary, the European populations are acknowledged as migratory [27]. But despite the very detailed information on their coevolutionary relationship with its main European corvid hosts during the breeding season (e.g. [29–34]), we only have fragmented and contradictory information regarding their migratory behavior. The low rate of ringing recoveries (0.4%, 8 out of 2012 marked individuals; [35]) only provides sparse information on the migratory pattern of its European individuals. Some authors propose them to be short-distance migrants [36] while others argue that they are long-distance migrants, with European birds crossing the Sahara to winter in West-Africa [37], where they are even able to reproduce [12]. No previous information regarding their stopovers or potential migratory routes exists. Adult cuckoos leave their Spanish breeding grounds in June [38], but it is not clear whether they initiate their migration immediately given that some marked individuals have been observed outside the breeding area in July [39]. This information is particularly important to understand the parent-offspring relationship in these brood parasites as traditionally it has been thought that juveniles and adults migrate separately, given that the former leave the Spanish breeding areas one month later than the latter [38]. This information would also be crucial to understand the development of migration abilities and whether juveniles rely on their parents’ experience or innate mechanisms [25]. Interestingly, it is not rare to observe both juveniles and adults in the same areas of West Africa [27]. Another important unknown aspect of its migratory behavior is the migratory strategy followed by this species. Observations of great spotted cuckoos in migration are very scarce [40,41], which could suggest that they are primarily solitary nocturnal migrants, like the common cuckoo [14], or that they do not use typical avian migratory routes (i.e. Strait of Gibraltar). In addition, no study has previously investigated the causes of mortality of adult great spotted cuckoos or its location, a type of information that is very scarce for animals in general [15,22]. Some studies found that the trans-Saharan migratory period is the most dangerous for (long-distance) migrants [21,22] while others propose that the majority of deaths occur in Europe [15]. This knowledge is critical to understand the fluctuations in their populations and consequently the pressure on their hosts.

The main aim of this study is to provide new knowledge on the movement ecology of one of the best-known brood parasitic species in order to expand our understanding on its ecology outside the well-known breeding period. Using satellite tracking devices, we investigate the annual migration cycle of adult great spotted cuckoos, their migratory strategy and the spatio-temporal distribution of their mortality. There are two different possibilities in relation to their migratory cycle: Spanish cuckoos will migrate to Western Africa either by crossing the Sahara (long-distance migrants; [37]) or not (short-distance migrants; [36]). Given that some individuals reproduce in the Sahel [12] we cannot discard the possibility that some individuals might choose to stay in Africa rather than return to Europe. Alternatively, breeding individuals in Africa could be part of a non-migratory population [19]. Regarding the initiation of migration, we would expect adult cuckoos to start their migration in June, just after the breeding period [27], or in July, if they stay in other non-breeding European areas [39]. The lack of knowledge on the migration of cuckoos based on traditional techniques (i.e. ringing; see above) could be partially explained if they do not use the Strait of Gibraltar to cross the Mediterranean Sea in their migration route. Finally, using the tracking devices and, for the first time, a new tool to find them in the field once the bird is dead (a goniometer; ARGOS), we expect to unveil the temporal and geographical components of the mortality of great spotted cuckoos. There is a growing concern on the illegal killing of birds along the southern Mediterranean shore [42,43], therefore, the majority of cuckoo deaths could be concentrated in Africa rather than in Europe. We explored the above-mentioned research questions for both male and female cuckoos in order to check for potential sex differences in their migratory behavior and performance.

## Materials and methods

### Field protocol

Using mist-nets and playback equipment broadcasting conspecific calls, we captured 16 adult great spotted cuckoos (10 males, 6 females) in southern Spain (Hoya de Guadix; 37°16’ N, 3°00’ W) during the breeding season (April-May) of 2013 and 2014. From each bird captured, we collected body weight measurements as well as a drop of blood from the brachial vein. The blood was stored in absolute ethanol in order to perform molecular sexing [44]. Each captured bird was ringed and equipped with a solar charged Argos Platform Terminal Transmitters (PTTs; Microwave Telemetry Inc.) attached as a backpack using a body harness made of 2mm flat-braided nylon cord [14,15]. The weight of the PTTs attached (5g) corresponded to an average 3.1% (2.8% - 3.4%) of the cuckoos’ body weight, below the recommended threshold for these devices [45]. Cuckoos were always released within 15 minutes after capture and flew away without apparent problems.

### Data preparation

Satellite transmitters were set to record in a 12h-on / 48h-off transmission cycle in 2013 and an 8h-on / 15h-off cycle in 2014. Geographical locations were obtained by the ARGOS/CLS service. Each Argos location fix is classified according to their accuracy based on the number of distinct messages received by the satellite and estimated error calculation (from greater to lower accuracy: 3–0, A-B or Z). In this study only high-quality fixes (3–1, accuracy: <250 m, <500 m, <1.5 km respectively) were used. The exception were 0 quality fixes (accuracy >1.5 km) during migration, which were used in locations where no higher quality fixes were available. Given the long distances involved and the scarcity of location data received during this time, these fixes still provide a good approximation of the birds’ location during this important part of the year. We manually removed two high accuracy fixes that were obviously incorrect after comparing their geographical and temporal information with those fixes from before and after. Our data were filtered to only include unique combinations of date, time and location using R 3.2.4 [46]. All fixes recorded within 5 min were removed except for the earliest fix in order to get a single representative of the location [47]. One PTT recorded reliable data for just two days (sixteen fixes) and data from this bird was not used in further analyses of migration strategy.

### Migration strategy

The breeding area was defined as the area around the capture site in the Hoya de Guadix (37°16’ N, 3°00’ W) up to 30 km. The migratory period lasted from the moment each bird left its final Spanish home range in a continuing southward direction until it reached the first location in the wintering area where it stayed for more than one full day. The wintering area was defined as the entire Sahel area south of the Sahara (18° North). For each location where multiple fixes were recorded within 15 km of each other, an average of the latitude and longitude coordinates was calculated, unless clear and distinct clusters could be distinguished (in areas of longer stay), or fixes were judged to be part of a longer journey to a new location based on visual inspection and the exact timing of fixes. Itineraries were created for each cuckoo by chronologically listing all (average) locations and the great-circle distance for each journey between subsequent locations (travelling segment) was calculated. The total migratory distance consisted of the sum of the distances of all travelling segments recorded throughout the migratory period. Since these segments consist of straight lines connecting the recorded locations and likely do not reflect the actual flight path of the cuckoos, distances are conservative estimates [14,15].

The duration of stay in each location and of each travelling segment was determined. Since PTTs did not always record a location every day, either because of the settings of the PTT or its inability to connect with a satellite [48], there were gaps in the data. When this occurred during a longer stay it was assumed that birds stayed in the same area although it cannot be ruled out that they made short trips to other areas. For the same reason exact dates of departure and arrival from each location were not always available. When this was the case, the duration of travel between locations was taken to reflect the maximum period during which travelling could have taken place. Consequently, the time spent in each location reflected minimum durations. The total duration of the actual migration was the exact time between the last recorded fix in the departure location and the first recorded fix in the wintering area during a stop longer than one day, minus the time spent in stopover sites. All stops longer than 2 days (including at least two transmission cycles) were classified as stopovers or staging areas.

Additionally, average travel speed per day was calculated by dividing the total migratory distance by the maximum number of travel days. Therefore, travel speeds reflect minima and could be faster if the number of travelling days was less than the maximum [49]. To see at what time of the day cuckoos prefer to travel, segments between pairs of high quality fixes recorded during the same transmission cycle were collected throughout the post-breeding migratory period (excluding stopovers). For each segment the travel speed was calculated. Following [49], a cut-off of 5 km/h was taken to determine if a bird was travelling or stationary. To exclude the effect of short, possibly local burst of activity, a minimum duration of 30 min per segment was set. In total 89 segments with a duration between 30 min and 5 h were collected. All segments occurring between 8 pm and 6 am GMT were classified as night-travel.

### Mortality data

Mortality of the tagged cuckoos was investigated after a PTT showed the same stationary location for at least 3 complete cycles. In such situation, we explored the temperature data recorded by the PTT. We assumed the cuckoo was dead when the tag’s temperature sensor showed diurnal fluctuations following environmental rhythm instead of being buffered by the body temperature of the bird [15]. Using the last position provided by the tag and a goniometer (CLS direction finder), we tried to locate the PTT in the field in order to estimate the potential cause of death. We attributed mortality to hunting when we found the nylon cord associated with the tag cut straight and one or several shotgun cartridges laying around it. When the nylon cord was torn and several (or most) cuckoo feathers were found in the area, we assumed the cuckoo was predated. We managed to retrieve 7 of the 16 tags. All recovered PTTs were working at the moment of retrieval. We used the last high-quality fix of the bird before the diurnal temperature rhythm started as the location of death, even if the tag was not found in the field [15]. We calculated cumulative daily mortality rates using four time periods (breeding/Spain, post-breeding migration, wintering, and pre-breeding migration). The length of each of these periods was calculated using average period durations adjusted to sum to 365 days.

### Molecular sexing

DNA was extracted from the blood using an ammonium acetate precipitation method following [50]. A small amount of blood cells was first air-dried before 250 μL proteinasing solution (0.2 mg/mL proteinase K, 50 mM Tris, 120 mM NaCl, 1% SDS, 20 mM EDTA, pH 8.0) was added. This mixture was digested for 3 hours at 55°C in a shaking heating block. This step results in destruction of the cells and removal of membrane lipids and proteins releasing the DNA from the cell nucleus. Subsequently, 250 μL ammonium acetate (4M) was added and the resulting solution vortexed for 15 min. The sample was centrifuged at 8000 g for 10 min before ±500 μL supernatant was transferred to a new tube and mixed with 1 mL EtOH. To precipitate the DNA from the supernatant the sample was centrifuged again at 8000 *g* for 10 min, the supernatant was taken out, and the pellet of DNA was rinsed with 300 μL 70% EtOH. The sample was again centrifuged at 8000 g for 5 min and the supernatant pipetted off and air-dried until all EtOH was evaporated. Finally, the isolated DNA samples were placed in 250 μL 10mM Tris, 0.1 mM EDTA and left overnight to dissolve the DNA.

PCR Amplification of CHD1 introns using the primers P2 and P8 [51], was used to determine cuckoo sex following [44]. One gene CHD-W is located on the W-chromosome and is thus exclusive to females while the other, CHD-Z, is located on the Z-chromosome and occurs in both sexes. The final PCR gel thus shows one band for males and two for females. The initial PCR mixture contained 3.0 mM MgCl2, 0.2 mM dNTP, 1x reaction buffer, 0.25 μM of each primer, 1U of Taq polymerase and 2 μL of genomic DNA as a template. Since this resulted in weak staining, a second PCR was run using Qiagen MP PCR mix, resulting in much clearer bands appearing on the gel. The thermal PCR cycle consisting of an initial 2 min denaturalization at 94°C; 30 cycles consisting of 45 s denaturalization at 94°C, 45 s annealing at 50°C, and 45 s extension at 72°C; ending with a final 2 min extension at 72°C. Of the sixteen birds, ten turned out to be males and six females.

### Statistical analyses

Regional differences in travel speed between locations were assessed using a Kruskal-Wallis Test, while post-hoc tests were performed using a pairwise Wilcoxon Rank Sum test. Wilcoxon Rank Sum test was also used to check for possible sex differences in migration speed. A chi-squared (*χ*^2^) test was used to test for differences between the proportions of day and night travel. Values are presented as mean ± standard deviation. All analyses were carried out using R 3.2.4 [46].

### Ethical statement

All research has been conducted according to relevant Spanish national (Real Decreto 1201/2005, de 10 de Octubre) and regional guidelines. The Consejería de Medio Ambiente of the Junta de Andalucía (Spain) approved this study and provided the corresponding permit (SGMN/GyB/JMIF).

## Results

One cuckoo was followed for three years and provided 4012 unique location fixes, the other fourteen birds were tracked between 12 days and 10 months (median = 75 days) providing between 114 and 2294 location fixes (median = 707). On average 46% (25–54%) of location fixes were of high quality (ARGOS classification 3–1) (S1 Table).

### Cuckoo migration route and wintering area

**Fig 1:**
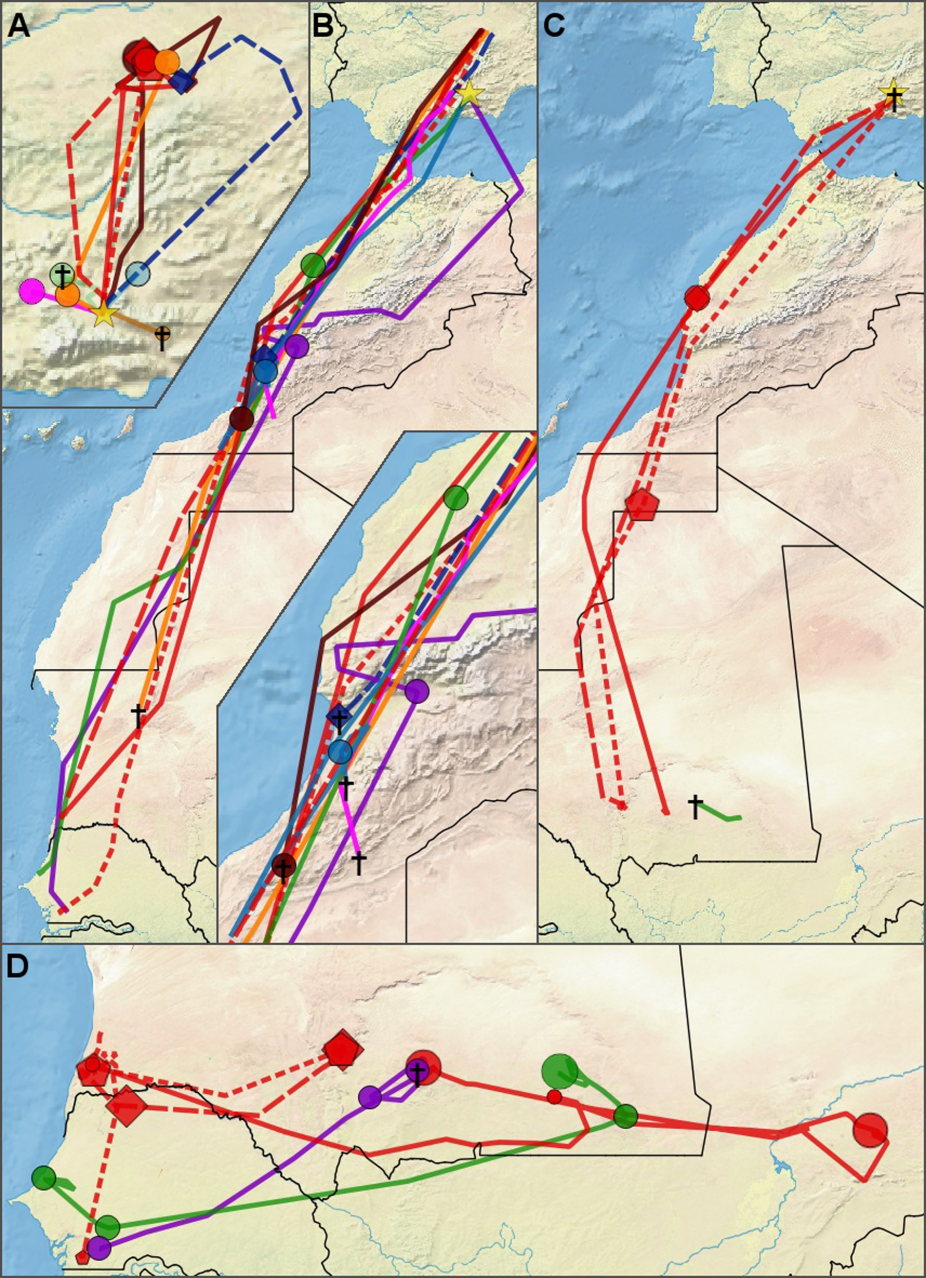
Map of migratory routes of great spotted cuckoos. **(A)** Pre-migratory movements within Spain. **(B)** Post-breeding migration **(C)** Pre-breeding migration **(D)** Wintering area movements. Colors correspond to individual cuckoos, only cuckoos that left the breeding area are included in this map. The breeding area is shown by the yellow star. Symbols are stopover or staging area locations with a larger size corresponding to a longer duration of stay. To increase clarity, only stopovers with a minimum duration of 7 days are included. Line types & symbols correspond to different years (2013: long dash / diamond, 2014: solid / circle, 2015: short dash / pentagon). Black crosses indicate locations where birds were lost (apparent mortality). For clarity, 7 mortality events that occurred within the breeding area were omitted. Lines connect recorded locations but might not reflect actual flightpaths.

Cuckoos showed variation in their post-breeding movements before migration, those carried out after the first cuckoo left the breeding area each year. Eight cuckoos left the breeding area for other regions in Spain (Fig 1). Surprisingly, four cuckoos travelled almost 200 km north to a region in the province of Albacete (Central Spain) where they stayed for 44 ± 16 days (mean ± s.d., n = 6). Five other cuckoos moved to nearby areas (<60 km away from the breeding grounds) while seven cuckoos did not leave the breeding area apart from occasional short trips of less than two days.

**Fig 2:**
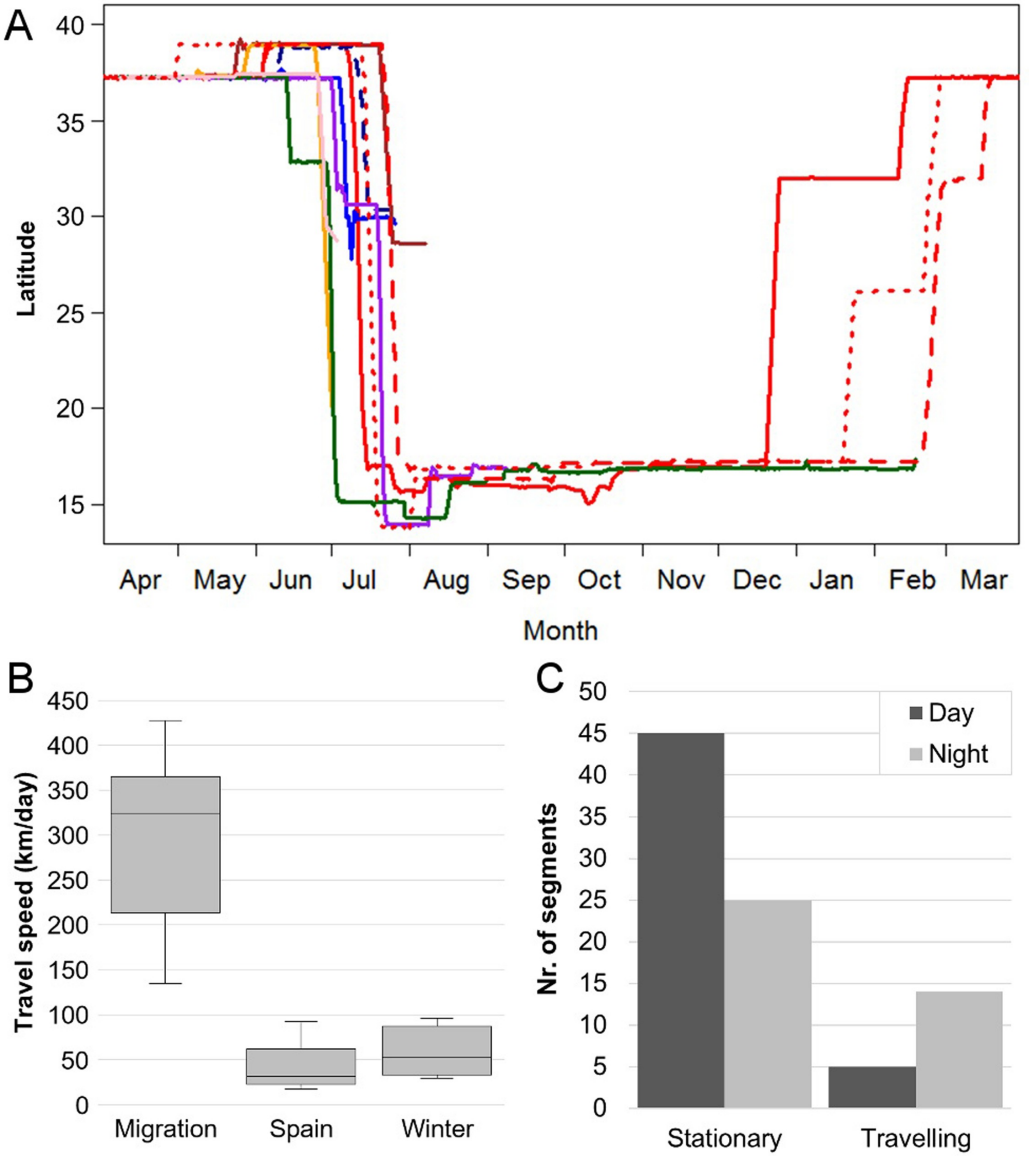
Migratory strategy. **(A)** Individual tracks of eight migratory great spotted cuckoos for latitude versus time. Colors correspond to individual cuckoos, line types correspond to different years (2013: dash, 2014: solid, 2015: dotted). Some birds travel north from the breeding area before initiating migration. Stopover sites in Morocco and wintering areas in sub-Saharan Africa can be distinguished. **(B)** Travel speed between locations within different regions. Boxplots show the median, first and third quartile ("hinges") and the minimum and maximum values ("notches"). **(C)** Total numbers of travel segments collected during migration classified as either stationary or travelling (speed of >5 km/h), occurring during either day or night. While the total number of travelling segments was lower than that of stationary segments, there were significantly more travelling segments recorded during the night.

Eight cuckoos initiated post-breeding migration in mid-June–July (Figs 2 and S1 and Table 1). three of them directly from the breeding area, one from a nearby area (55 km) and four from Central Spain. The other eight cuckoos were lost prior to migration (S1 Table). There was no indication of cuckoos staying in the breeding area or even in Spain year-round. The routes of all but one bird suggest that cuckoos cross the western Alboran Sea or the strait of Gibraltar into Morocco. Five cuckoos made a stopover in southern Morocco lasting 13.7 ± 1.2 days, whereas two others continued uninterrupted.

**Table 1:** Migratory strategy of the great spotted cuckoo. In the calculation of means only birds that completed migration were included, except for average travel speed which includes all birds apart from Green in spring migration since that bird died just a day after initiating migration. Cuckoo names correspond to the colors used in other figures. † indicates that bird was lost during migration. Brown and Dark Blue were lost during the stopover.

	*Cuckoo*	*Year*	*Sex*	*Distance (km)*	*Migratory period*	*Stopover duration (days)*	*Max*. *travel days*	*Av*. *travel speed (km/day)*
**Post-breeding**	Red	2013	M	2733	21 Jul—27 Jul	-	6.4	427
	Red	2014	M	2847	7 Jul—15 Jul	-	8.8	324
	Red	2015	M	3170	12 Jul—21 Jul	-	9.1	348
	Orange #2	2014	M	2370†	24 Jul—3 Jul†	-	7.3†	325†
	Purple	2014	M	3475	1 Jul—23 Jul	12.1	10.0	348
	Brown	2014	M	1392†	20 Jul—7 Aug†	13.0†	4.5†	309†
	Dark blue	2013	F	1128†	11 Jul—31 Jul†	15.8†	6.6†	171†
	Blue	2014	F	1729†	4 Jul—26 Jul†	13.5	7.6†	228†
	Pink	2014	F	1157†	26 Jun—3 Jul†	-	8.6†	135†
	Green	2014	F	2899	13 Jun—4 Jul	14.0	7.0	414
	***Mean ± s*.*d*.**			***3025 ± 267***		***13*.*2 ± 0*.*8***	***8*.*3 ± 1*.*3***	***303 ± 92***
**Pre-breeding**	Red	2014	M	2619	19 Feb—18 Mar	13.6	15.1	173
	Red	2015	M	2634	21 Dec—14 Feb	46.8	8.6	306
	Red	2016	M	2517	20 Jan—27 Feb	27.4	11.3	223
	Green	2014	F	161†	16–17 Feb†	-	2.1†	77†
	***Mean ± s*.*d*.**			***2590 ± 52***		***29*.*3 ± 13*.*6***	***11*.*7 ± 2*.*7***	***234 ± 55***

Three cuckoos successfully crossed the Sahara and reached the wintering grounds 13.2 ± 6.9 days after starting migration (n = 5). All birds initially travelled towards Southwest Mauretania or Senegal and stayed there for several days to months (43 ± 27 days; n = 5), before moving inland and spending the majority of the winter in south (east) Mauretania (145 ± 30 days; n = 4). Since in some occasions cuckoos spent almost three months in the coastal region, and both coastal and inland areas were located at similar latitudes, they were both considered to be wintering grounds. The five birds that reached the wintering grounds did so in 8.3 ± 1.3 travelling days (excluding stopover time) covering 3025 ± 267 km with a speed of 372 ± 41 km/day (Table 1).

During their stay in the wintering grounds, all birds showed clear itinerancy, staying in 7–9 different locations for 12.1 ± 9.5 days in each (n = 31). The longest stay always occurred in the final location (76.9 ± 33.8 days, n = 4). Distances travelled during this period varied greatly 1547 ± 811 km, n = 4; Table 2). However, the migratory spread or the distance between the final wintering areas was relatively small (226.3 ± 142.6 km).

**Table 2:** Spatial behavior of the great spotted cuckoo in Spain and the wintering area. In the calculation of means only birds that survived the entire stage were included, except for average travel speed which includes all birds. Cuckoo names correspond to the colors used in other figures. † indicates that bird was lost during that period.

*Cuckoo*	*Year*	*Sex*	*Distance (km)*	*Period*	*# Stops*	*Stopover duration & range (days)*	*Max*. *travel days*	*Travel speed*
**Spain**								
Red	2013	M	311	2 May—21 Jul	4	17.3 (4.7–44.1)	10.7	29
Red	2014	M	243	19 Mar—6 Jul	2	51.3 (27.0–75.6)	6.6	37
Red	2015	M	221	14 Feb—12 Jul	3	46.5 (26.8–73.0)	8.8	25
Dark blue	2013	F	378	4 May—9 Jul	3	18.4 (7.0–25.1)	10.8	35
Light blue	2013	M	222	13 May—26 Jun†	5†	6.3 (2.5–11.8)	12.8†	17
Light green	2013	F	314	13 May—13 Jun†	3†	15.9 (9.5–23.2)	13.4†	23
Orange #1	2013	M	49	14 May—2 Jul†	2†	23.3 (7.1–39.5)	2.2†	22
Orange #2	2014	M	278	29 Apr—24 Jun	3	17.1 (9.4–25.4)	4.5	62
Yellow	2014	M	97	9 Apr—24 May†	2†	21.8 (20.3–23.3)	1.8†	54
Purple	2014	M	13	29 Apr—1 Jul	2	31.2 (22.2–40.2)	0.7	19
Blue	2014	F	173	29 Apr—4 Jul	3	19.7 (1.7–36.3)	7.2	24
Brown	2014	M	435	8 Apr—20 Jul	4	24.7 (2.7–46.4)	4.7	93
Pink	2014	F	58	8 Apr—25 Jun	2	38.4 (30.5–46.3)	0.8	73
Lime	2014	M	622	8 Apr—12 Jun†	6†	9.7 (2.0–15.6)	9.8†	64
***Mean ± s*.*d*.**			***244 ± 161***	***2*.*9 ± 0*.*7***		***24*.*4 ± 12*.*6***	***6*.*1 ± 3*.*6***	***41 ± 23***
**Wintering Area**								
Red	2013	M	691	27 Jul—18 Feb	7	26.0 (2.3–88.8)	23.7	29
Red	2014	M	2818	15 Jul—20 Dec	9	10.5 (2.2–43.1)	53.4	53
Red	2015	M	1029	23 Jul—19 Jan	9	17.0 (2.5–43.2)	27.4	38
Purple	2014	M	995†	23 Jul—9 Sep†	3†	12.6 (10.2–16.3)	10.3†	97
Green	2014	F	1649	5 Jul—15 Feb	8	20.4 (3.8–118.7)	21	79
***Mean ± s*.*d*.**			***1547 ± 811***	***8*.*3 ± 0*.*8***		***17*.*3± 5*.*5***	***31*.*4 ± 12*.*9***	***59* ± *25***

### Migratory strategy

Travel speed varied significantly between different periods of the year (*χ*^2^ = 20.082, df = 2, p < 0.0001). The average migratory travel speed between locations was 303 ± 92 km/day (including the birds that died during migration, n = 10). This was significantly faster than travel speed between locations measured in Spain (41.2 ± 22.7 km/day, p < 0.0001, n = 14) and the wintering area (58.9 ± 25.2 km/day, p = 0.0013, n = 5; Fig 2B). Investigating the exact timing of post-breeding migration revealed that cuckoos prefer to travel at night, with a significantly larger proportion (73.7%) of travelling segments occurring between 8 pm and 6 am (*χ*^2^ = 6.7, df = 1, p = 0.009; n = 19; Fig 2C). Not enough travelling segments were available for the pre-breeding migration to assess whether cuckoos are also nocturnal travelers during this period.

### Intra-individual differences

Since one bird (Red, a male) provided data for three consecutive migratory cycles, intra-individual differences could be observed. Red made a stopover in Morocco during each pre-breeding migratory journey but never during post-breeding migration like some other cuckoos. His average pre-breeding migration distance of 2590 ± 52 km was remarkably consistent and was on average 327 km shorter than his post-breeding migration distance (which includes the additional 200 km from starting their migratory route from further north in Central Spain). Red’s pre-breeding migration (11.7 ± 2.7 days) took slightly longer than his post-breeding migration (8.1 ± 1.2 days). However, more gaps of missing data during pre-breeding migration (9.2 days) than for post-breeding migration (4.0 days) made it more difficult to determine exact travelling dates, resulting in slower pre-breeding than post-breeding migration (234 ± 55 km/day vs. 366 ± 44 km/day).

Red showed both consistencies and differences in the destinations and timing of migration between years (Figs 1 and 2 and S1). He visited the same areas in Central Spain and south-west Mauretania each year also visiting the same final wintering location and pre-breeding stop-over site twice. However, there were also clear differences, especially in 2014 when he traveled much further east and left the wintering area almost two months earlier compared to other years.

### Cuckoo mortality

**Fig 3:**
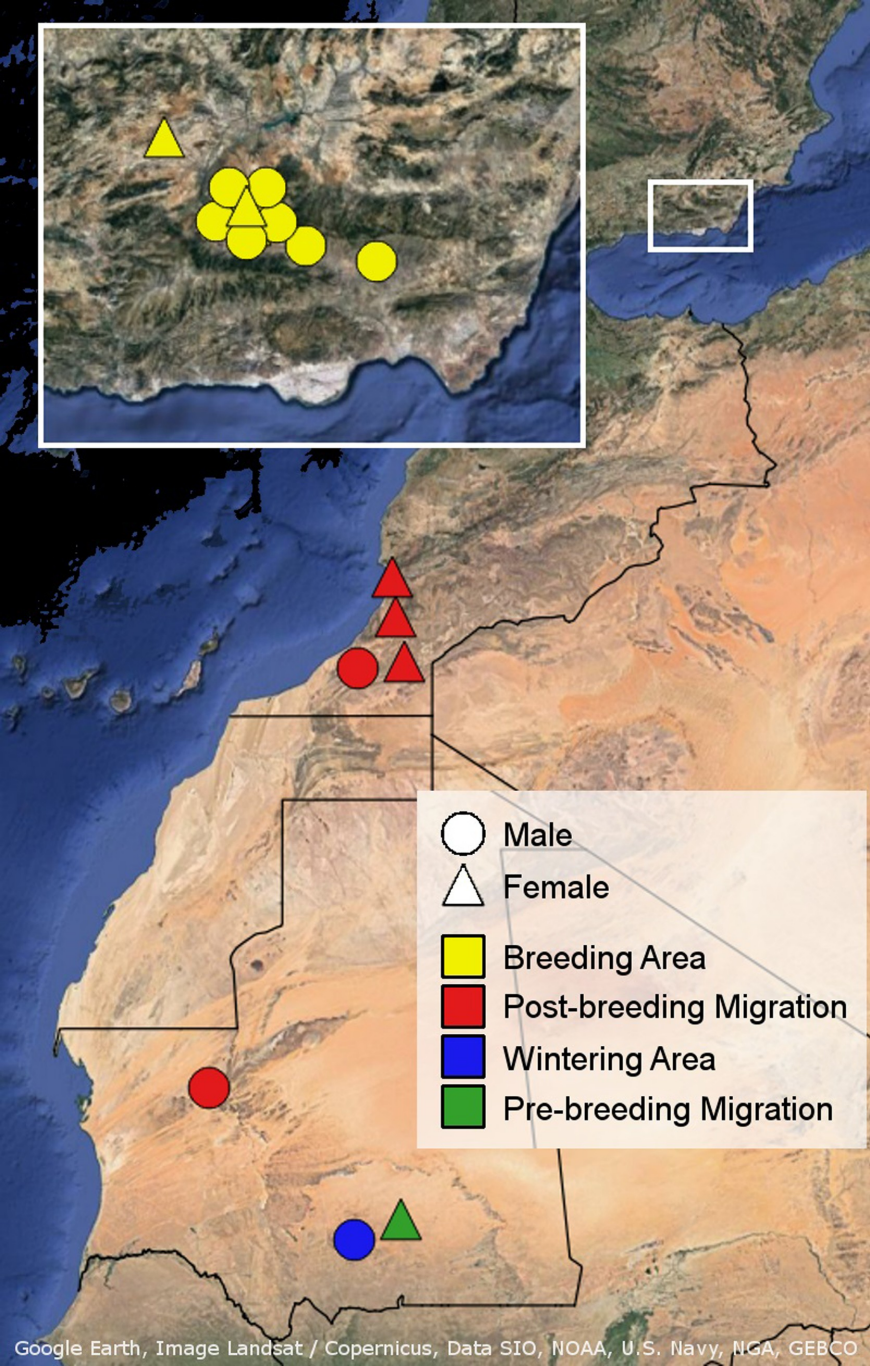
Mortality map. Apparent mortality locations of tracked male and female cuckoos.

Mortality data showed that most cuckoos were lost in Europe (n = 9) and in southern Morocco (n = 4; Fig 3). Out of the nine cuckoos that died in Europe, seven died in the breeding area and two in areas close by (<60 km). In total, eight died before initiating migration. Five cuckoos were lost during the first post-breeding migratory period: three while travelling and two during a stopover. These deaths occurred in southern Morocco, apart from one travelling bird that died in Mauretania. One cuckoo was lost after a month in the wintering area in southern Mauretania, another in the Mauritanian Sahara two days after initiating pre-breeding migration in February. Since the duration of the migratory period was much shorter than the duration of time spent in the breeding area, the daily mortality rate was more than 7 times higher during migration (0.0452 during pre-breeding migration, 0.0062 in Spain). In Europe, predation was the biggest cause of death (n = 4/5 known causes), while one bird died of unknown causes, possibly weather related. For birds that died in south Morocco during the migratory period, the cause of death could only be determined for two birds: one died from predation and the other was shot.

### Sex differences

**Fig 4:**
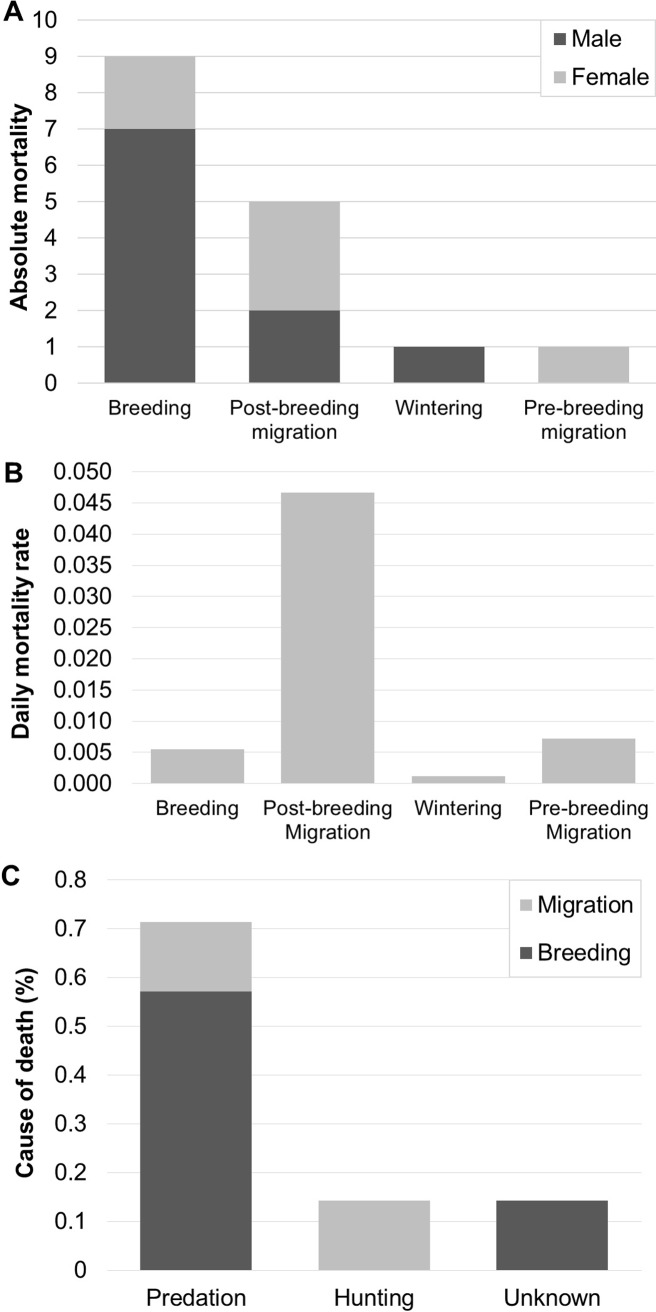
Number of tracked cuckoos that died during each period of the annual cycle. **(A)** Total number of deaths of each sex. **(B)** Daily mortality rate. **(C)** Causes of death for recovered cuckoos in the breeding area and during migration.

There were no indications for sex differences in migratory behavior. Cuckoos of both sexes stayed or left the breeding area, went to the same locations if they left it, and migrated towards the same regions in sub-Saharan Africa. The observed difference in migratory speed (♂: 347 ± 38, ♀: 237 ± 108 km/day; n = 10) was not statistically significant either (W = 5, p = 0.17). Additionally, there were no indications for differences in the distance and timing of migration or the preferred travel time of day, although these comparisons were not tested statistically due to the low sample sizes. Regarding mortality, we found that the majority of males died in Europe (70%; n = 10), and only two died during migration. For females the opposite pattern was found with most deaths occurring during migration (67%; n = 6) and only two in Europe (Fig 4A).

## Discussion

In this study we provide, for the first time, complete information on the migratory behavior and performance of great spotted cuckoos, one of the best known brood parasites [33].

### Migration route

We have confirmed that great spotted cuckoos breeding in the south of Spain are long-distance migrants supporting previous assumptions [27,37]. Great spotted cuckoos breeding in southern Spain cross the Western Sahara Desert using the East Atlantic Flyway on their post-breeding migratory route (Fig 1B). Although the majority of them stop in Morocco for several days, none of the tracked individuals winter before crossing the Sahara Desert, which contradicts the short-distance migration hypothesis [36]. Interestingly, this population uses a similar route during their pre-breeding migration (Fig 1C) which contrasts with the loop strategy used by most common cuckoos [14,15]. The wintering grounds were always in the Sahel region, expanding from the coastal areas of Senegal to the Inner Niger Delta in Mali, which is still far from the Nigerian location where cuckoos were reported to be reproducing in Africa [12]. This would match with previous suggestions indicating that, in addition to the main breeding cuckoo populations in Southern Europe, Middle East and South Africa, there is a resident cuckoo population breeding in Central Africa [19]. It is however also possible that birds that breed in other areas in Europe (i.e. southern France or Italy) migrate to other regions in the Sahel (i.e. northern Nigeria). New studies tracking other European or African great spotted cuckoo populations will provide valuable insights in order to distinguish between these alternative options and understand the connectivity between populations.

Our individual-based study provides a surprising finding regarding the migratory route of this species, as we found that several individuals travel 200 km north, to the same region in central Spain, when they leave their ‘typical’ breeding area (Figs 1A and 2A). It has been traditionally thought that adult great spotted cuckoos initiate their migration when they leave their breeding areas [27]. But recent observations questioned this assumption, as some marked individuals were observed in nearby regions in Spain after the breeding period [39]. We confirmed that great spotted cuckoos can follow both strategies. We found that 37.5% of marked cuckoos start their migration directly from the breeding grounds, while the majority of them (62.5%) initiated it from other locations in Spain far from the place where they bred.

There could be several reasons why cuckoos go to other regions before initiating migration: (i) They might breed in those areas too, particularly cuckoos that travel north, as the breeding period of their corvid hosts could be delayed at more northern latitudes. Although we think this is an unlikely possibility given that the main breeding season of black-billed magpies (their main hosts) in Central Spain seem to be finishing when cuckoos visit those areas [52]; (ii) They could be prospecting territories for future breeding attempts. This is an intriguing possibility that requires further study, however, existing evidence suggests that host information from the same breeding season might play a larger role than that obtained in the previous season. For instance, cuckoos arrive to Spain very early in the season, well before their corvid hosts have started to build their nests [39], and nest size and egg color play a determinant role in host selection in the magpie-cuckoo system [53,54]; (iii) They could seek out areas with higher food availability in order to refuel their depleted energy reserves after the breeding period in order to maximize their probability of surviving the migration period. In other birds such as raptors, similar pre-migratory movements have been argued to be the result of short-term regional variation in food availability [55–57]. Body fat reserves have been demonstrated to be critical to survive migration in other species (e.g. [58]), particularly in those using few stopovers and covering long distances in each step [59], like the great spotted cuckoo. These non-mutually exclusive hypotheses have important implications for the co-evolutionary relationship between cuckoos and their hosts, and should be investigated in future studies.

The fact that some cuckoos stay in the breeding area while others leave indicates the existence of different individual strategies that certainly requires further research, but that already allows speculation. Those individuals that stay in their breeding grounds could provide some previously unknown parental care behaviors to their fledglings. In fact, there is some evidence that shows adult cuckoos and fledglings interacting in the study area, which seems to be important for the correct imprinting in this species [60]. Alternatively, these birds might be using public information to gather knowledge on the territory quality of their magpie hosts. This strategy however has been suggested to be limited to the current breeding season for obligate brood parasites [61], whereas its use for subsequent breeding seasons seems to be associated to facultative parasites [62].

### Migratory strategy

We found that great spotted cuckoos, like common cuckoos [63,64], are primarily nocturnal migrants (Fig 2C). Our data suggest that they are solitary migrants too, as none of the marked individuals traveled together or in the exact same period (Fig 2A). However, due to the relatively small number of marked individuals, we cannot rule out the possibility that our cuckoos were traveling with other unmarked birds. This question is of particular importance for two reasons. First, the suggestion of them being solitary migrants gives some insight into the mating system of cuckoos, as males and females do not seem to stay together when not breeding (even if captured at the same time in the same exact moment; e.g. Red and Dark blue, Purple and Blue; S1 Table). Genetic analyses indicate that the mating system of this species is very plastic varying from monogamy to polygamy, probably depending on population density [31,65]. Thus, a solitary migration strategy, in addition to the low adult survival probability (see below), could imply that cuckoos establish new pairs every year favoring the plasticity in their mating system. Second, if cuckoos are solitary migrants, then the discussion of whether fledglings travel alone or not [39] and the hypothesis that they might provide some parental care during migration (i.e. guiding fledglings to the wintering grounds) seems unlikely.

The annual migration strategy of great spotted cuckoos generally consists of five stages (Fig 2A): A relatively long stay in their breeding grounds (*c*. 4 months); a short stay of one additional month either in their breeding areas or in other non-breeding areas in Spain; a short (1–3 weeks) post-breeding migration period which might or might not contain a stopover; a very long stay (*c*. 6 months) in the wintering grounds in the Sahel; and a pre-breeding migration period of variable length depending mainly on stopover duration (2–5 weeks). This migration cycle differs from the one used by common cuckoos, which is composed of four stages, initiating their migration directly from their breeding grounds [14]. The average travelling speed during migration is similar to that found for the common cuckoo [14], although the total distance travelled is much shorter.

### Mortality causes and location

We found that the post-breeding migratory period, and particularly the south of Morocco, seems to be the most dangerous stage and area for cuckoos as they hold the highest daily mortality rate (Figs 3 and 4). Our results match with previous studies indicating the importance of the migratory period in investigations of mortality in birds [15,22,66]. However, the number of deaths that occurred during the breeding period was also very high indicating that fluctuations in European great spotted cuckoo populations would be influenced by mortality both in Europe and the north of Africa. Curiously, the wintering grounds seem to be a relatively safe area, although this conclusion should be taken with caution due to the small sample size of marked individuals that reached sub-Saharan Africa.

To our knowledge, we provide the first evidence for the causes of mortality of a long-distance medium-sized migratory bird using longitudinal data. We found that natural predation seems to be the major cause of death in this species (Fig 4C). Some evidence indicates the relevance of raptors as the main predator for this species. We have observed a peregrine falcon (*Falco peregrinus*) successfully hunting a great spotted cuckoo in our study area (J.D. Ibáñez-Álamo pers. obs.). Furthermore, the presence of either peregrine or barbary falcons (*F*. *pelegrinoides*) in the locations where the satellite tracking devices were recovered, as well as finding the feathers plucked or in a raptor feeding spot, point to the activity of a raptor (S2 Fig). In fact, great spotted cuckoos have been found among the prey items of several raptor species in Spain like the booted eagle (*Hieraaetus pennatus*), the black kite (*Milvus migrans*) or the Eastern imperial eagle (*Aquila heliaca*) [67–69]. Interestingly, we found that hunting activity also could be an important source of mortality during migration (Fig 4C). There are some records of great spotted cuckoo captures using trammel nets in Egypt [43] and, for instance, illegal killing is recognized as a significant threat to bird species in the Mediterranean [42]. Nevertheless, in Spain, which holds the largest number of great spotted cuckoos in Europe, the species does not seem to be very affected by these mortality factors as populations seem to be increasing [28]. Particularly interesting would be to investigate whether mortality patterns in great spotted cuckoos parasitizing (or raised by) different hosts could vary. Cuckoos in our study area mainly parasitize black-billed magpies (*Pica pica*), but also carrion crow (*Corvus corone*) [70,71]. Alternatively, and considering that cuckoos can also reproduce in the Sahel [12], African populations could act as sources too. This possibility however seems unlikely given the genetic structure of the European cuckoo populations which indicates a pattern of isolation by distance [32]. Future studies in our and other breeding populations would provide much needed information regarding these questions.

### Sex differences

Our study is, to our knowledge, the first offering long-term information not restricted to the breeding area for both male and female brood parasites as previous studies on the common cuckoo have been focused exclusively on males (e.g. [14–16,24]). No sex differences were detected regarding the migratory behavior, although a larger sample size of tracked individuals would be needed to fully explore this possibility, particularly regarding their movements in the wintering areas and pre-breeding migration. On the contrary, our results suggest that the most dangerous area for each sex differs: the breeding area seems to be more risky for males than for females. An increased mortality risk of males during breeding has been attributed to conspicuous competitive behaviors, such as mate searching or fighting, which increase the probability of predation [72]. Our finding that the majority of deaths in the breeding area were due to predation (Fig 4C) is in line with this hypothesis.

### Conclusions

To sum up, we provide new information on the migratory behavior and performance of great spotted cuckoos. We found evidence that suggests that cuckoos breeding in southern Spain are nocturnal long-distance migrants that winter in a large area in the Sahel region and use the East Atlantic Flyway on both their post and pre-breeding migratory routes. We recorded that cuckoos could follow two different strategies once they finish breeding, to stay in the breeding area or to leave for other areas in Spain, indicating important individual variation in their migratory strategy that requires further research. Contrary to the traditional idea, adult cuckoos were found to not initiate their migration directly after breeding, but to migrate south at similar dates as fledglings. In addition, we identified the south of Morocco as likely the most dangerous area for this brood parasite and that the main cause of death is natural predation, probably by raptors. This study shows the benefit of including long-term individual data obtained with satellite tracking technology in order to expand our knowledge not only on the ecology of brood parasites, but also regarding its co-evolutionary interactions with the hosts.

## Supporting information

S1 Fig Post-breeding migratory routes. Individual post-breeding migratory routes for those cuckoos that crossed the Mediterranean sea. Line types & symbols correspond to different years (2013: long dash / diamond, 2014: solid / circle, 2015: short dash / pentagon).
(TIF)

S2 Fig Predated great spotted cuckoo. Remains of a great spotted cuckoo found with the Goniometer attributed to predation by raptor. Area were the remains were found (1), specific raptor feeding spot (2) and close up of the PTT associated to it (3).
(JPG)

S1 Table Summary information for great spotted cuckoos. (PDF)

S2 Table Database. (CSV)
