# Supplementary figures and images for: Migration behavior and performance of the great spotted cuckoo (Clamator glandarius)
Source: PLoS One. 2019 Jan 4;14(1):e0208436. doi: 10.1371/journal.pone.0208436 (PMC6319774; doi:10.1371/journal.pone.0208436)

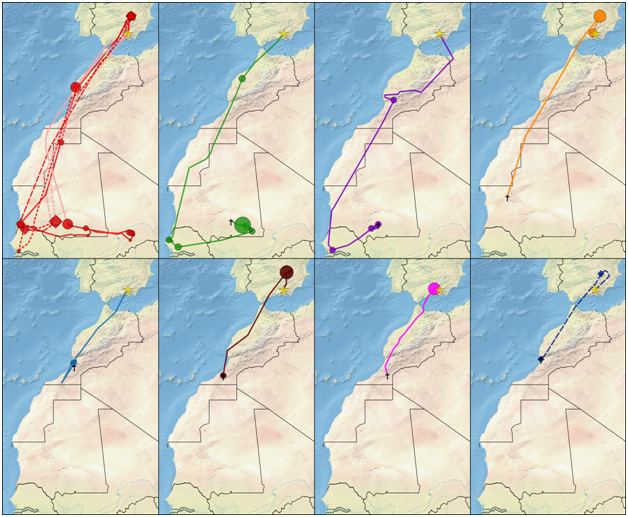

Supplement: S1 Fig — Individual post-breeding migratory routes for those cuckoos that crossed the Mediterranean sea. Line types & symbols correspond to different years (2013: long dash / diamond, 2014: solid / circle, 2015: short dash / pentagon). (TIF) [file pone.0208436.s001.tif]

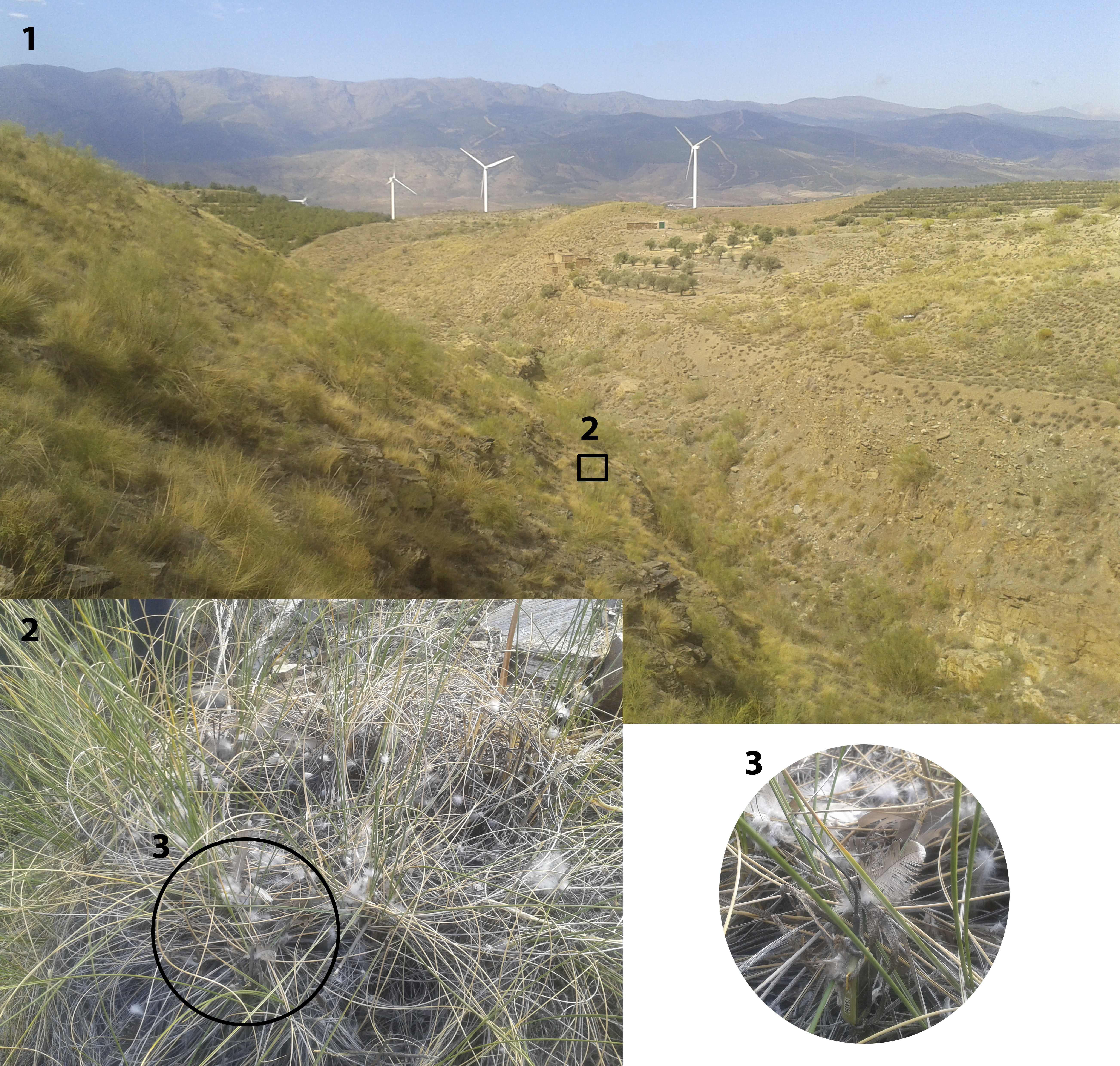

Supplement: S2 Fig — Remains of a great spotted cuckoo found with the Goniometer attributed to predation by raptor. Area were the remains were found (1), specific raptor feeding spot (2) and close up of the PTT associated to it (3). (JPG) [file pone.0208436.s002.jpg]
