# Supplementary material for: Migration behavior and performance of the great spotted cuckoo (Clamator glandarius)
Source: PLoS One. 2019 Jan 4;14(1):e0208436. doi: 10.1371/journal.pone.0208436 (PMC6319774; doi:10.1371/journal.pone.0208436)
Supplement: S1 Table — (PDF) [file pone.0208436.s003.pdf]

Table S1. Summary information for each tracked great spotted cuckoo.

Name	*PTT Nr*	*Year*	*Sex*	*# Fixes*	*%HQ Fixes*	*# days tracked*	*Weight (g)*	*Notes*
Red	123619	2013	M	4012	56.2%	1093	161.7	Migrated 3x, lost in breeding area
Dark Blue	123620	2013	F	460	47.0%	88	-	Migrated, lost during migration
Light Blue	123621	2013	M	246	49.2%	44	161.9	Lost before migration
Light Green	123622	2013	F	405	44.7%	61	165.4	Lost before migration
Orange #1	123623	2013	M	337	39.8%	49	150.4	Lost before migration
Orange #2	123623*	2014	M	287	52.6%	65	177.6	Migrated, lost during migration
White	132110	2014	F	749	47.8%	82	175.0	Lost before migration
Black	132111	2014	M	114	40.4%	11	156.5	Lost before migration
Yellow	132112	2014	M	416	44.0%	45	158.6	Lost before migration
Purple	132113	2014	M	1215	50.7%	133	168.3	Migrated, lost in wintering area
Blue	132114	2014	F	1025	25.3%	88	163.9	Migrated, lost during migration
Brown	134356	2014	M	1278	44.4%	121	161.7	Migrated, lost during migration
Dark Yellow~	134357	2014	M	16	100.0%	2	168.8	Lost before migration
Pink	134358	2014	F	1217	49.7%	86	171.7	Migrated, lost during migration
Lime	134359	2014	M	707	54.2%	68	148.9	Lost before migration
Green	134360	2014	F	2294	42.7%	316	157.0	Migrated, lost in wintering area
		Median (exl. Red):		**707 (584)**	**45.9%**	**82 (75)**	**163.5**	

*~ = not included in average, * = reused recovered PTT, - = not measured*
